# Genetic interaction between central pair apparatus genes *CFAP221, CFAP54,* and *SPEF2* in mouse models of primary ciliary dyskinesia

**DOI:** 10.1038/s41598-020-69359-3

**Published:** 2020-07-23

**Authors:** Casey W. McKenzie, Lance Lee

**Affiliations:** 1 Pediatrics and Rare Diseases Group, Sanford Research, 2301 E. 60th Street N., Sioux Falls, SD 57104 USA; 2 Department of Pediatrics, Sanford School of Medicine of the University of South Dakota, 1400 W. 22nd Street, Sioux Falls, SD 57105 USA

**Keywords:** Genetics, Diseases, Pathogenesis

## Abstract

Primary ciliary dyskinesia (PCD) is a genetically heterogeneous syndrome that results from defects in motile cilia. The ciliary axoneme has a 9 + 2 microtubule structure consisting of nine peripheral doublets surrounding a central pair apparatus (CPA), which plays a critical role in regulating proper ciliary function. We have previously shown that mouse models with mutations in CPA genes *CFAP221*, *CFAP54*, and *SPEF2* have a PCD phenotype with defects in ciliary motility. In this study, we investigated potential genetic interaction between these CPA genes by generating each combination of double heterozygous and double homozygous mutants. No detectable cilia-related phenotypes were observed in double heterozygotes, but all three double homozygous mutant lines exhibit early mortality and typically develop severe PCD-associated phenotypes of hydrocephalus, mucociliary clearance defects in the upper airway, and abnormal spermatogenesis. Double homozygous cilia are generally intact and display a normal morphology and distribution. Spermiogenesis is aborted in double homozygotes, with an absence of mature flagella on elongating spermatids and epididymal sperm. These findings identify genetic interactions between CPA genes and genetic mechanisms regulating the CPA and motile cilia function.

## Introduction

Primary ciliary dyskinesia (PCD) is a syndrome resulting from dysfunction of motile ciliary clearance in the respiratory system, the brain, the fallopian tube, and the embryonic node, as well as sperm flagellar motility^1–5^. It is genetically heterogeneous and commonly inherited in an autosomal recessive manner, although mutations causing X-linked recessive and autosomal dominant inheritance have been reported^6–9^. Approximately 1 in 16,000 children are affected and typically exhibit chronic upper and lower airway infection, otitis media, male infertility, and situs inversus. Neonatal respiratory distress, congenital heart defects, female infertility, and hydrocephalus are also associated at a lower frequency. The core, or axoneme, of the motile cilium has a 9 + 2 microtubule structure with nine doublets along the outer periphery surrounding a central pair apparatus (CPA)^4,10^. Ciliary motility is generated by dynein arms associated with the outer microtubule doublets and is regulated by the CPA, radial spokes connecting the outer and central microtubules, and the dynein regulatory complex. The cilia on the embryonic node possess a 9 + 0 microtubule structure without a CPA.

A substantial number of mouse models of PCD has helped uncover the role of novel genes in ciliary function and PCD pathogenesis^4,5^. However, only a few studies have investigated epistatic interaction of these genes. Mice lacking dynein assembly factor coiled coil domain containing protein 40 (CCDC40) have situs inversus with randomized expression of left–right patterning marker *NODAL* during gastrulation^11,12^. Homozygous mutants that are also heterozygous for a *NODAL* mutation fail to establish left isomerism or left-sided expression of *NODAL*^12^, demonstrating that these genes interact to drive left sidedness in the gastrulating embryo*.* Mutations in the retinitis pigmentosa GTPase regulator (RPGR) gene result in PCD phenotypes with an associated retinitis pigmentosa in humans and mouse models^8,13–15^. Mice lacking RPGR were crossed to a line with a mutation in the gene encoding centrosomal protein 290 (CEP290), resulting in double homozygotes with a more severe and rapid progression of retinal degeneration, indicating a genetic interaction between *RPGR* and *CEP290*^16^. Finally, homozygous loss of CPA genes sperm associated antigen 6 (*SPAG6*) and sperm associated antigen 16L (*SPAG16L*) produce very different phenotypes. *SPAG6* mutants exhibit the full spectrum of PCD phenotypes, CPA structural defects, and reduced ciliary beat frequency^17–19^, while mice lacking *SPAG16L* have only spermatogenic defects and male infertility but no other cilia-associated phenotypes^20^. However, a genetic interaction was identified in double homozygous mutants, which have hydrocephalus and abnormal airway pathology that are more severe than the *SPAG6* homozygotes alone^21^. Despite the paucity of studies investigating genetic interactions, the phenotypes of double mutant mice uncover new information about the roles of these genes in motile cilia function.

**Figure 1:**
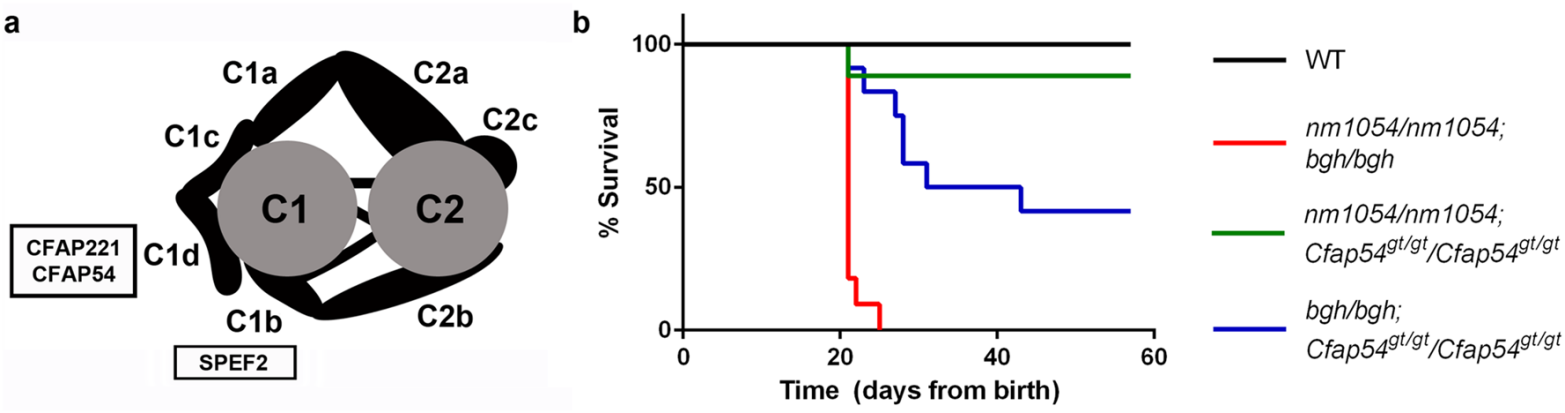
Early mortality of mice lacking two CPA genes. (**a**) Schematic diagram of the CPA showing the location of CFAP221, CFAP54, and SPEF2, which are absent in the *nm1054, Cfap54*^*gt/gt*^, and *bgh* mouse lines, respectively. Modified with permission from McKenzie et al.^29^. (**b**) Survival curve showing mortality of *nm1054/nm1054;bgh/bgh*, *nm1054/nm1054;Cfap54*^*gt/gt*^/*Cfap54*^*gt/gt*^, and *bgh/bgh;Cfap54*^*gt/gt*^/*Cfap54*^*gt/gt*^ double homozygotes prior to adulthood.

The CPA is a complex structure that regulates ciliary motility and waveform^22–25^. It is comprised of several protein complexes, or projections, that associate with the core microtubules. We have previously shown that mouse models with mutations in CPA genes ciliary and flagellar associated protein 221 (*CFAP221*), ciliary and flagellar associated protein 54 (*CFAP54*), and sperm flagellar protein 2 (*SPEF2*) each have a PCD phenotype that includes hydrocephalus, male infertility, and airway abnormalities^26–30^. The PCD in mice homozygous for the *nm1054* mutation, a large deletion that removes *CFAP221*, also known as primary ciliary dyskinesia protein 1 (*PCDP1*), results from cilia with reduced ciliary beat frequency (CBF) but no detectable ultrastructural abnormalities^26^. The *Chlamydomonas reinhardtii* homolog of CFAP221 associates with the C1d projection, a calcium-dependent CPA complex required for proper flagellar motility^31–33^. A gene trapped allele of *CFAP54*, which also encodes a member of the C1d projection complex in *C. reinhardtii*^31–33^, results in reduced CBF, perturbed cilia-driven fluid flow, and loss of the C1d projection^29^. The ultrastructural defect observed in cilia from mice lacking CFAP54 indicates that CFAP54 and CFAP221 are functionally distinct despite both proteins associating with the same complex in *C. reinhardtii*. Finally, the spontaneous *big giant head* (*bgh*) mouse line has a nonsense mutation in *SPEF2* that, like mice lacking CFAP221, does not result in axonemal ultrastructural defects but does reduce the CBF^27^. Unlike CFAP221 and CFAP54, however, SPEF2 localizes to the C1b projection in *C. reinhardtii* rather than the C1d projection^34^. Figure 1a shows a schematic diagram of the CPA and indicates the location of the CFAP221, CFAP54, and SPEF2 proteins. Since nodal cilia lack a CPA, none of these models develop the laterality defects often associated with PCD. Recently, *CFAP221* and *SPEF2* mutations were identified in human PCD patients^35–37^, highlighting the importance of these CPA genes.

Here, we investigate potential genetic interaction between *CFAP221, SPEF2,* and *CFAP54* by crossing the *nm1054, bgh,* and *Cfap54*^*gt/gt*^ mouse lines. Double heterozygotes show no detectable PCD phenotypes, but the *nm1054/nm1054;bgh/bgh, nm1054/nm1054;Cfap54*^*gt/gt*^*/Cfap54*^*gt/gt*^*,* and *bgh/bgh;Cfap54*^*gt/gt*^*/Cfap54*^*gt/gt*^ double homozygotes all exhibit early mortality and often severe hydrocephalus and airway phenotypes. Double homozygous cilia are generally intact with a normal morphology and distribution. Severe defects in spermatogenesis were also observed, although marker expression analysis suggests that some ciliary components are still being assembled. These data underscore the critical role of CPA proteins in regulating proper motile cilia function and unveil genetic interactions between CPA genes.

## Results

### Double homozygosity results in early lethality

Mice homozygous for the *nm1054*, *Cfap54*^*gt/gt*^, and *bgh* mutations lack CPA projection proteins CFAP221, CFAP54, and SPEF2, respectively (Fig. 1a). Each single mutant was shown to exhibit early mortality on the C57BL/6J (B6) background due to severe hydrocephalus but typically live a normal life span on the 129S6/SvEvTac (129) background or a mixed background without gross hydrocephalus^26,27,29^. In contrast, early mortality was common for double homozygotes on a mixed background (Fig. 1b). The most severe mortality phenotype was observed in *nm1054/nm1054;bgh/bgh* double mutants, with all mice dying or requiring euthanasia due to severe hydrocephalus by weaning age (3 weeks). The *bgh/bgh;Cfap54*^*gt/gt*^/*Cfap54*^*gt/gt*^ double homozygotes also demonstrated substantial mortality, with less than half surviving to adulthood (8 weeks). Survival was best for *nm1054/nm1054;Cfap54*^*gt/gt*^/*Cfap54*^*gt/gt*^ double mutants, with most mice surviving to adulthood. Gross hydrocephalus was common in mice that died prior to 8 weeks, regardless of genotype. All double heterozygotes (*nm1054/*+ *;bgh/*+ *, nm1054/*+ *;Cfap54*^*gt/gt*^/+ , and *bgh/*+ *;Cfap54*^*gt/gt*^*/*+) survived to adulthood and were analyzed at 8 weeks or older, along with wild type (WT) controls (Supplementary Table S1).

### Double homozygosity results in severe hydrocephalus

**Figure 2:**
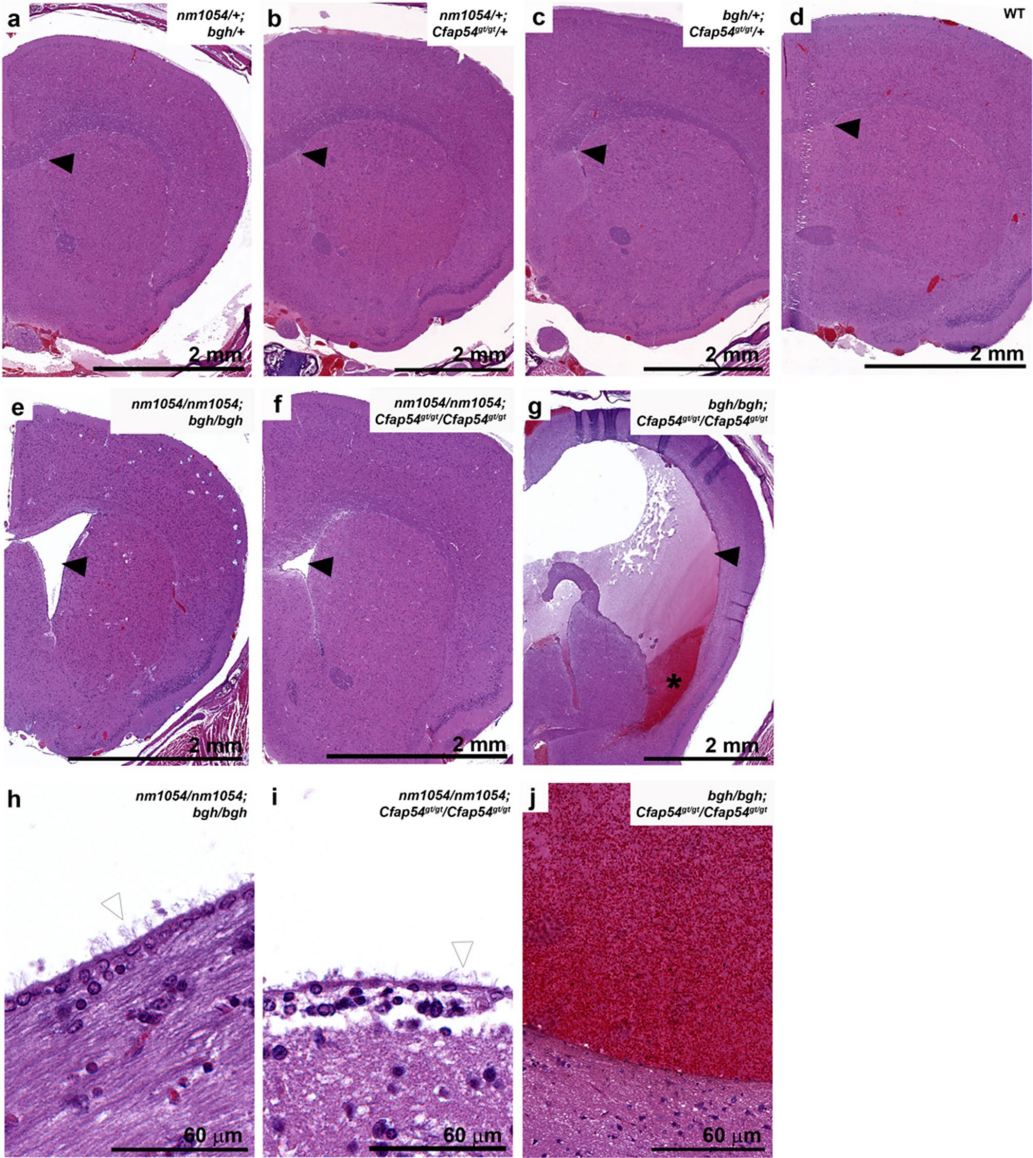
Histological analysis of double mutant brains. Coronal sections of double heterozygous (**a**–**c**), WT (**d**), and double homozygous (**e**–**j**) brains showing dilatation of the lateral ventricles in double homozygotes. Sections are stained with H&E. Closed arrowheads indicate the lateral ventricle, open arrowheads indicate ependymal cilia, and the asterisk (*) indicates blood inside the lateral ventricle.

To assess the extent of damage in the brains of double mutant mice on a mixed background, we performed histological analyses on coronal sections through the lateral ventricles, through which cerebrospinal fluid (CSF) flows and accumulates under hydrocephalic conditions. The lateral ventricle is appropriately narrow in WT and double heterozygous mice with no evidence of tissue damage (Fig. 2a–d). However, a severe hydrocephalic phenotype was observed in all three double homozygotes (Supplementary Fig. S1). The *nm1054/nm1054;bgh/bgh* brain shows enlarged ventricles but not extensive tissue damage, and ependymal cilia lining the ventricular wall are intact (Fig. 2e,h). It is important to note, however, that only one mouse survived long enough for analysis (Fig. 1b), so the phenotype may have been more severe in those animals that died earlier. The *nm1054/nm1054;Cfap54*^*gt/gt*^/*Cfap54*^*gt/gt*^ double homozygotes typically showed mild ventricular dilatation without extensive tissue damage, and ependymal cilia remained intact (Fig. 2f,i). The most severe phenotype was observed in *bgh/bgh;Cfap54*^*gt/gt*^/*Cfap54*^*gt/gt*^ brains, which commonly exhibited severe ventricular dilatation, as well as extensive damage to the white matter and cerebral cortex that was often accompanied by intraventricular hemorrhaging with a dramatic influx of red blood cells (Fig. 2g,j). While *nm1054, bgh,* and *Cfap54*^*gt/gt*^ single mutants were each shown to exhibit severe hydrocephalus, this phenotype was only observed on the more susceptible B6 background and not on a mixed background^26,27,29^.

### Double homozygosity results in severe airway abnormalities

**Figure 3:**
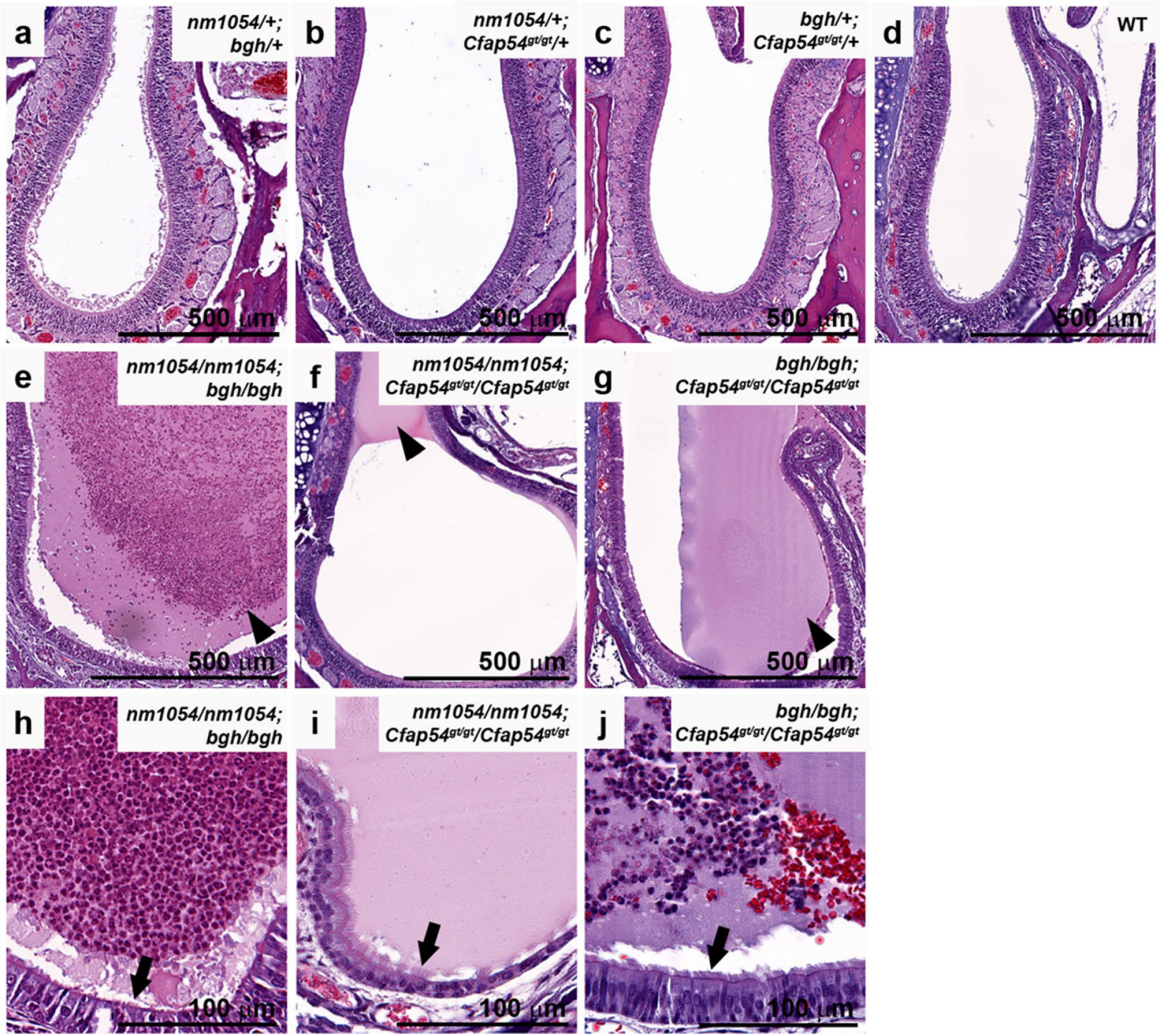
Histological analysis of double mutant sinus cavities. Coronal sections of double heterozygous (**a**–**c**), WT (**d**), and double homozygous (**e**–**j**) maxillary sinus cavities showing mucus accumulation and neutrophil infiltration in double homozygotes. Sections are stained with H&E. Arrowheads indicate mucus with or without neutrophils, and arrows indicate epithelial cilia.

Since motile ciliary dysfunction typically results in airway abnormalities, we assessed the pathology of the maxillary sinus cavity. WT and double heterozygous mice have a clear sinus cavity with little to no evidence of mucus (Fig. 3a–d). In contrast, all three double homozygotes exhibit a more severe defect in mucociliary clearance and airway pathology (Supplementary Fig. S1). The one surviving *nm1054/nm1054;bgh/bgh* mouse shows severe accumulation of mucus and extensive infiltration of neutrophils, which is common in sinusitis pathology and is indicative of the tissue repair process (Fig. 3e,h). Despite the mucus and white blood cell accumulation, some motile cilia remain intact on the epithelial surface (Fig. 3h). The *nm1054/nm1054;Cfap54*^*gt/gt*^/*Cfap54*^*gt/gt*^ double homozygotes typically showed some areas of mild mucus accumulation but no evidence of extensive neutrophil infiltration, and epithelial cilia remain intact (Fig. 3f,i). Like the *nm1054/nm1054;bgh/bgh* mouse, the *bgh/bgh;Cfap54*^*gt/gt*^/*Cfap54*^*gt/gt*^ sinus cavity typically shows severe accumulation of mucus and areas with mild infiltration of neutrophils, sometimes accompanied by red blood cells (Fig. 3g,j). Motile cilia remain largely intact on *bgh/bgh;Cfap54*^*gt/gt*^/*Cfap54*^*gt/gt*^ sinus epithelia (Fig. 3j). The sinus phenotype in double homozygous mice is consistent with mucus accumulation in the single *nm1054, bgh,* and *Cfap54*^*gt/gt*^ mutants on a mixed background, as well as neutrophil infiltration in *bgh* mice, but the presence of red blood cells was never observed in single mutants^26,27,29^.

**Figure 4:**
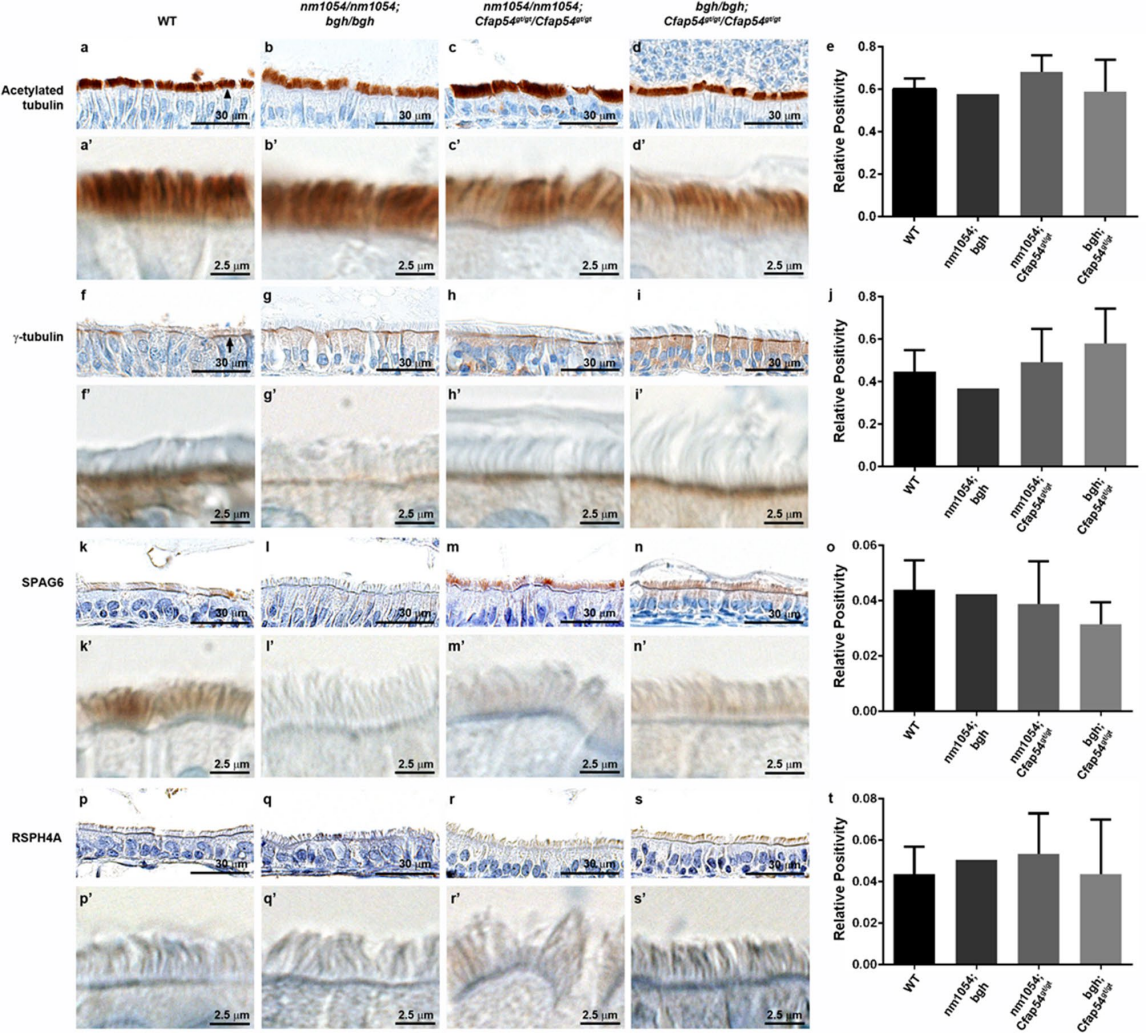
Immunohistochemical analysis of ciliary markers in double homozygous sinus. Sections of WT, *nm1054/nm1054;bgh/bgh*, *nm1054/nm1054;Cfap54*^*gt/gt*^/*Cfap54*^*gt/gt*^, and *bgh/bgh;Cfap54*^*gt/gt*^/*Cfap54*^*gt/gt*^ maxillary sinus airway epithelia stained with antibodies to ciliary marker acetylated tubulin (**a**–**d**,**a**′–**d**′), basal body marker γ-tubulin (**f**–**i**,**f**′–**i**′), CPA marker SPAG6 (**k**–**n**,**k**′–**n**′), and radial spoke marker RSPH4A (**p**–**s**,**p**′–**s**′). The arrowhead (**a**) indicates the motile cilia, and the arrow (**f**) indicates the basal bodies. Quantification of acetylated tubulin, γ-tubulin, SPAG6, and RSPH4A staining intensity is shown in (**e**,**j**,**o**,**t**, respectively). nm1054;bgh: *nm1054/nm1054;bgh/bgh*, nm1054;Cfap54^*gt/gt*^: *nm1054/nm1054;Cfap54*^*gt/gt*^/*Cfap54*^*gt/gt*^, bgh: Cfap54^*gt/gt*^: *bgh/bgh;Cfap54*^*gt/gt*^/*Cfap54*^*gt/gt*^. All p values are relative to WT. For acetylated tubulin (**e**), p = 0.479 for *nm1054/nm1054;Cfap54*^*gt/gt*^/*Cfap54*^*gt/gt*^ and 0.977 for *bgh/bgh;Cfap54*^*gt/gt*^/*Cfap54*^*gt/gt*^ mice. For gamma tubulin (**j**), p = 0.871 for *nm1054/nm1054;Cfap54*^*gt/gt*^/*Cfap54*^*gt/gt*^ and 0.357 for *bgh/bgh;Cfap54*^*gt/gt*^/*Cfap54*^*gt/gt*^ mice. For SPAG6 (**o**), p = 0.772 for *nm1054/nm1054;Cfap54*^*gt/gt*^/*Cfap54*^*gt/gt*^ and 0.253 for *bgh/bgh;Cfap54*^*gt/gt*^/*Cfap54*^*gt/gt*^ mice. For RSPH4A (**t**), p = 0.804 for *nm1054/nm1054;Cfap54*^*gt/gt*^/*Cfap54*^*gt/gt*^ and 0.999 for *bgh/bgh;Cfap54*^*gt/gt*^/*Cfap54*^*gt/gt*^ mice. Statistical significance was determined by one-way ANOVA. Since only one *nm1054/nm1054;bgh/bgh* mouse survived for tissue collection, no statistical analysis was performed.

The morphology and distribution of motile cilia lining the sinus cavity was further assessed by immunohistochemistry (IHC). Ciliary marker acetylated tubulin is expressed normally in WT cilia (Fig. 4a,a′), while basal body marker γ-tubulin localizes to the ciliary base (Fig. 4f,f′). Staining of acetylated tubulin confirms that the intact cilia on all three double homozygotes have a generally typical morphology and distribution consistent with WT mice, and there is no statistically significant difference in acetylated tubulin levels (Fig. 4b–e,b′–d′). In addition, γ-tubulin is normally expressed and properly localized to the ciliary base in all three double homozygotes, suggesting that ciliogenesis is not perturbed (Fig. 4g–j,g′–i′). Known CPA protein SPAG6 is expressed throughout the axoneme of WT cilia (Fig. 4k,k′), and relatively weak SPAG6 staining in cilia from double homozygous sinus epithelia suggests that defects in proper CPA assembly are possible, although there are no statistically significant differences (Fig. 4l–o,l′–n′). Analysis of known radial spoke marker RSPH4A shows little difference in axonemal expression or protein level between WT and double homozygous mice (Fig. 4p–t,p′–s′), further confirming a largely normal ciliary assembly. Consistent with the normal pathology of the sinus cavity in double heterozygous mice, the morphology and distribution of motile cilia appears normal in all three double heterozygotes, also with no statistically significant difference in acetylated tubulin levels (Supplementary Fig. S2). In addition, tracheal CBF was measured for double heterozygotes using a high speed video microscopy approach, and levels were not statistically different from WT for any of the double heterozygous mice, confirming that the motile cilia are functionally normal (Supplementary Fig. S3). CBF analysis could not be performed efficiently or accurately on double homozygotes due to the low number of mice surviving to adulthood and technical challenges associated with measurement using the small neonatal or young postnatal tracheae.

### Double homozygosity impairs spermatogenesis

**Figure 5:**
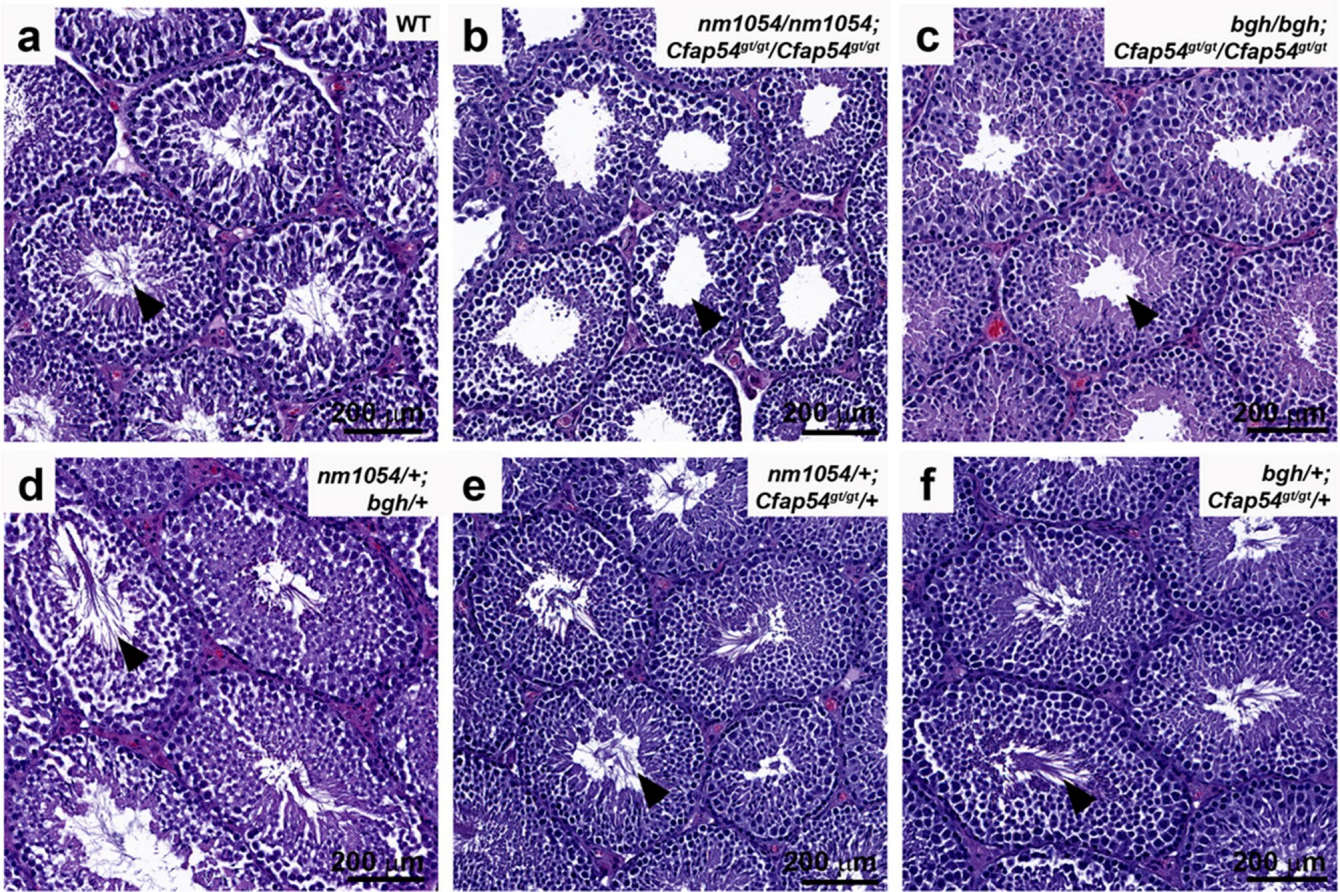
Histological analysis of double mutant testes. Sections of WT (**a**), double homozygous (**b**,**c**), and double heterozygous (**d**–**f**) testes showing an absence of elongating spermatid flagella in double homozygotes. Sections are stained with H&E. Arrowheads indicate the lumen of the seminiferous tubule, where elongating spermatid flagella are generated during spermiogenesis.

Although male mice lacking CFAP221, SPEF2, or CFAP54 are infertile^26,27,29^, fertility tests were not performed for double homozygotes due to the low number of mice surviving to sexual maturity. Double heterozygotes are fertile and were used routinely for breeding to obtain double homozygotes. Effect of double homozygosity on spermatogenesis was investigated using a histopathological approach. As elongating spermatids develop into mature spermatozoa during spermiogenesis, the final step of spermatogenesis, WT flagella extend into the lumen of the seminiferous tubule of the testis (Fig. 5a). In contrast, *nm1054/nm1054;Cfap54*^*gt/gt*^/*Cfap54*^*gt/gt*^ and *bgh/bgh;Cfap54*^*gt/gt*^/*Cfap54*^*gt/gt*^ double homozygotes both show an absence of flagella in the lumen of the seminiferous tubule (Fig. 5b,c), indicating that flagellar formation is perturbed. While this is different from the presence of motile cilia on the sinus epithelia from these mice, it is not dissimilar to the spermatogenesis phenotype observed in single *nm1054, bgh,* or *Cfap54*^*gt/gt*^ mutants^26,27,29^. No testis data was obtained for *nm1054/nm1054;bgh/bgh* double homozygotes, as none survived to sexual maturity, and the only one that survived long enough to collect tissues was a female. Normal spermiogenesis was observed in the testes from *nm1054/*+ *;bgh/*+ *, nm1054/*+ *;Cfap54*^*gt/gt*^/+ , and *bgh/*+ *;Cfap54*^*gt/gt*^*/*+ double heterozygous mice (Fig. 5d–f), which is consistent with their fertility.

**Figure 6:**
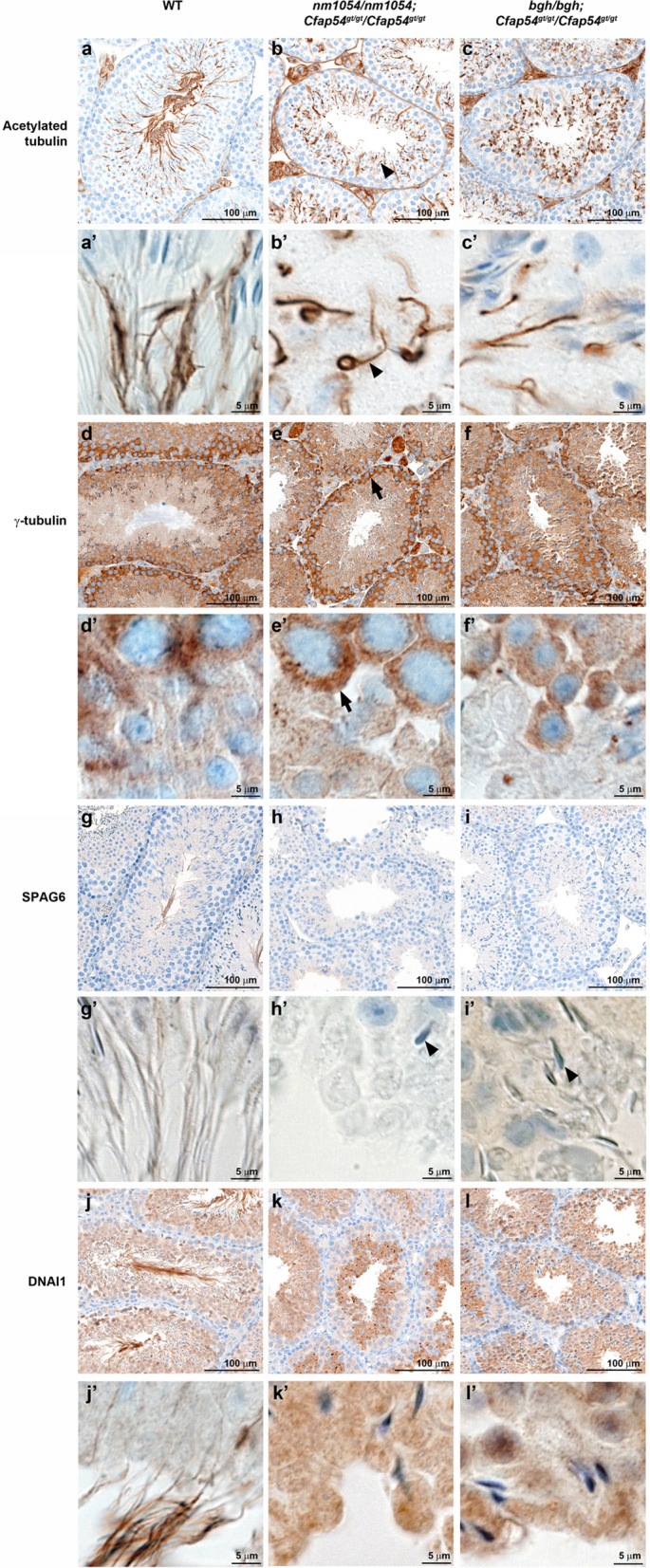
Immunohistochemical analysis of ciliary markers in double homozygous testes. Sections of WT, *nm1054/nm1054;Cfap54*^*gt/gt*^/*Cfap54*^*gt/gt*^, and *bgh/bgh;Cfap54*^*gt/gt*^/*Cfap54*^*gt/gt*^ testis stained with antibodies to flagellar marker acetylated tubulin (**a**–**c**,**a**′–**c**′), basal body marker γ-tubulin (**d**–**f**,**d**′–**f**′), CPA protein SPAG6 (**g**–**i**,**g**′–**i**′), and dynein protein DNAI1 (**j**–**l**,**j**′–**l**′). The arrowheads indicate rudimentary axonemal structures (**b**,**b**′) and developing sperm heads (**h**′,**i**′), and the arrows indicate the spermatogonium cell type (**e**,**e**′).

To further investigate the ability of double homozygous testes to assemble flagella, we performed IHC analysis. An antibody to ciliary and flagellar marker acetylated tubulin effectively detects the elongating flagella in the lumen of the WT seminiferous tubule (Fig. 6a,a′). While no flagella are present in the lumen of *nm1054/nm1054;Cfap54*^*gt/gt*^/*Cfap54*^*gt/gt*^ or *bgh/bgh;Cfap54*^*gt/gt*^/*Cfap54*^*gt/gt*^ double homozygous testes, there are defined structures detected by the acetylated tubulin antibody in the developing spermatids from both mutants (Fig. 6b,c,b′,c′), suggesting that some rudimentary axonemal structures possessing acetylated tubulin may be assembled in those cells. The antibody to basal body marker γ-tubulin primarily stains immature spermatogonia and developing spermatocytes within the WT seminiferous tubule and both double homozygotes (Fig. 6d–f,d′–f’′), suggesting that early stages of spermatogenesis are unaffected. However, while CPA protein SPAG6 is detected throughout the flagellar axoneme in WT seminiferous tubules (Fig. 6g,g′), low SPAG6 levels without a clear staining pattern in double homozygotes suggests that CPA assembly may be impaired (Fig. 6h,h′,i,i′). The defined axonemal structures detected by the acetylated tubulin antibody in *nm1054/nm1054;Cfap54*^*gt/gt*^/*Cfap54*^*gt/gt*^ and *bgh/bgh;Cfap54*^*gt/gt*^/*Cfap54*^*gt/gt*^ spermatids are not detected by the SPAG6 antibody, with only occasional sperm heads observed. An antibody to known dynein marker DNAI1 also stains the length of the flagellar axoneme in WT testis (Fig. 6j,j′). In contrast to SPAG6, DNAI1 shows strong expression in the developing spermatids surrounding the lumen of the *nm1054/nm1054;Cfap54*^*gt/gt*^/*Cfap54*^*gt/gt*^ and *bgh/bgh;Cfap54*^*gt/gt*^/*Cfap54*^*gt/gt*^ seminiferous tubules (Fig. 6k,k′,l,l′), indicating that dynein pre-assembly may occur in the spermatid cytoplasm even in the absence of mature flagella. Acetylated tubulin staining in *nm1054/*+ *;bgh/*+ *, nm1054/*+ *;Cfap54*^*gt/gt*^/+ , and *bgh/*+ *;Cfap54*^*gt/gt*^*/*+ double heterozygotes highlights their morphologically normal elongating spermatid flagella (Supplementary Fig. S4).

**Figure 7:**
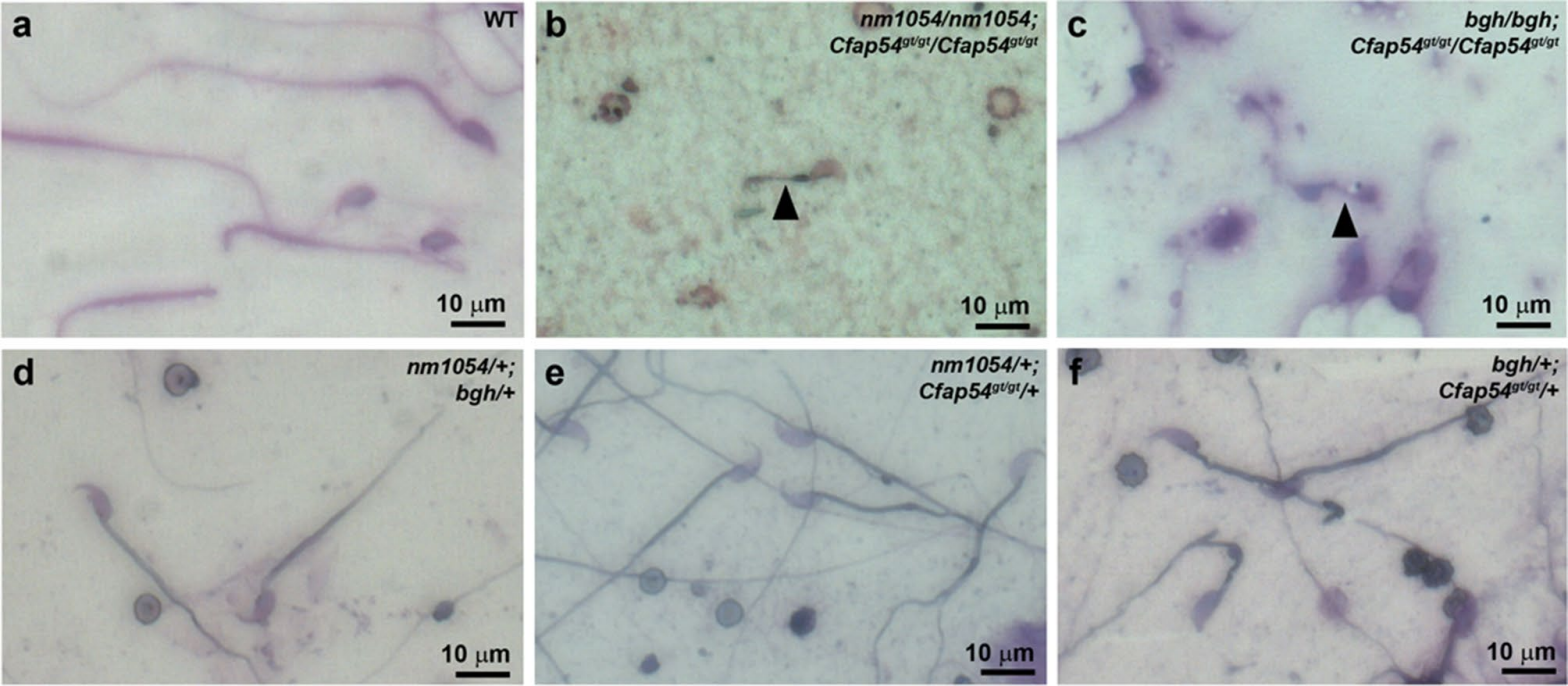
Morphology of double mutant sperm. Epididymal sperm from WT (**a**), double homozygous (**b**,**c**), and double heterozygous (**d**–**f**) mice showing shortened flagella on double homozygous sperm. Sections are stained with the Camco differential stain kit. Arrowheads indicate the shortened flagella on double homozygous sperm.

In addition to testis pathology, the morphology of mature sperm from the epididymis was also assessed using light microscopy. WT sperm have a hook-shaped head and a long flagellum (Fig. 7a). In contrast, sperm were rarely present in the *nm1054/nm1054;Cfap54*^*gt/gt*^/*Cfap54*^*gt/gt*^ or *bgh/bgh;Cfap54*^*gt/gt*^/*Cfap54*^*gt/gt*^ double homozygous epididymis, and those that were present have normal heads but severely shortened flagellar stubs that would likely impair proper sperm motility (Fig. 7b,c). As described above, sperm data could not be obtained for *nm1054/nm1054;bgh/bgh* double homozygotes, as only one female mutant survived long enough to collect tissues. Consistent with the testis data and normal fertility, all double heterozygous mice have epididymal sperm that are indistinguishable from WT sperm (Fig. 7d–f).

## Discussion

In this study, we investigated potential genetic interaction between CPA genes *CFAP221, SPEF2,* and *CFAP54* by crossing the *nm1054*, *bgh,* and *Cfap54*^*gt/gt*^ mouse models with mutations in those genes. We identified an absence of ciliary phenotypes in double heterozygotes, but *nm1054/nm1054;bgh/bgh, nm1054/nm1054;Cfap54*^*gt/gt*^*/Cfap54*^*gt/gt*^*,* and *bgh/bgh;Cfap54*^*gt/gt*^*/Cfap54*^*gt/gt*^ double homozygotes all exhibit PCD phenotypes of hydrocephalus, mucociliary clearance defects, and spermatogenesis abnormalities. Double mutants also exhibit severe early mortality on a mixed background that is not observed in single mutants. Double homozygous cilia appear intact and normally distributed, with the exception of the ependymal cilia, where the severe ventricular dilatation observed in *bgh/bgh;Cfap54*^*gt/gt*^*/Cfap54*^*gt/gt*^ double mutants can cause extensive tissue damage. In addition, spermiogenesis is aborted in double homozygous males, and there is an absence of mature flagella on elongating spermatids and epididymal sperm.

The absence of histopathological or ciliary phenotypes in any of the double heterozygous mice (*nm1054/*+ *;bgh/*+ *, nm1054/*+ *;Cfap54*^*gt/gt*^*/*+ *,* and *bgh/*+ *;Cfap54*^*gt/gt*^*/*+) indicates that haploinsufficiency for two different CPA genes does not result in a ciliary phenotype. While this is expected given the autosomal recessive inheritance associated with these mutations, it demonstrates that a dose-dependent reduction in two different CPA proteins does not put a significant enough strain on the CPA to noticeably affect its function or the function of the motile cilium. The PCD phenotypes associated with the double homozygous mutants, however, are striking and generally more severe than single mutants. Most notable is the early mortality associated with double homozygotes on a mixed genetic background (Fig. 1). Homozygotes with either the *nm1054, bgh,* or *Cfap54*^*gt/gt*^ mutation exhibit early mortality on the B6 background due to severe hydrocephalus, presumably due to genetic modifiers segregating in that strain that influence susceptibility to severe hydrocephalus^26,27,29,30,38^. On the 129 background or a mixed background, single homozygotes do not develop severe hydrocephalus and live a normal life span, despite exhibiting airway ciliary phenotypes and male infertility. All three double homozygotes (*nm1054/nm1054;bgh/bgh*, *nm1054/nm1054;Cfap54*^*gt/gt*^/*Cfap54*^*gt/gt*^, and *bgh/bgh;Cfap54*^*gt/gt*^/*Cfap54*^*gt/gt*^) develop hydrocephalus on a mixed background (Fig. 2), suggesting that the defect in CSF flow in double homozygotes may be primarily due to severe ciliary dysfunction and highlighting genetic interactions. Given that several double homozygotes had to be euthanized in the early post-natal period due to severe hydrocephalus, the hydrocephalus is the most likely cause of the early mortality.

Despite the presence of motile cilia in the brain and airway of double homozygous mutants, there is an absence of mature flagella on elongating spermatids and epididymal spermatozoa from *nm1054/nm1054;Cfap54*^*gt/gt*^/*Cfap54*^*gt/gt*^ and *bgh/bgh;Cfap54*^*gt/gt*^/*Cfap54*^*gt/gt*^ double homozygous mutants (Figs. 5, 7). Reproductive data was not obtained for *nm1054/nm1054;bgh/bgh* double homozygotes due to mortality prior to sexual maturity. The difference between motile cilia and sperm flagella is consistent with single *nm1054, bgh,* and *Cfap54*^*gt/gt*^ homozygous mutants, as well as mutations in other axonemal genes that prevent proper flagellar formation without affecting ciliogenesis^4,26,27,29,39^. Presence of acetylated tubulin staining in developing spermatids from *nm1054/nm1054;Cfap54*^*gt/gt*^/*Cfap54*^*gt/gt*^ and *bgh/bgh;Cfap54*^*gt/gt*^/*Cfap54*^*gt/gt*^ double homozygotes suggests that the spermatids start to make axonemes, but the process aborts early in spermiogenesis (Fig. 6). In addition, DNAI1 staining in spermatids also indicates that cytoplasmic dynein pre-assembly is occurring in the double homozygotes. However, low staining without a clear staining pattern for CPA marker SPAG6, along with absence of detectable axonemal structures, suggest that CPA assembly may be perturbed. These data underscore fundamental differences in the mechanisms that drive and regulate motile ciliogenesis and sperm flagellar formation.

Interestingly, the *nm1054/nm1054;Cfap54*^*gt/gt*^/*Cfap54*^*gt/gt*^ double homozygous phenotype is noticeably less severe than the phenotypes of *nm1054/nm1054;bgh/bgh* and *bgh/bgh;Cfap54*^*gt/gt*^/*Cfap54*^*gt/gt*^ double homozygotes. The *nm1054/nm1054;Cfap54*^*gt/gt*^/*Cfap54*^*gt/gt*^ double mutants exhibit a much higher survival rate (Fig. 1), show mild ventricular enlargement in the brain (Fig. 2, Supplementary Fig. S1), and display only modest amounts of mucus accumulation in the sinus cavity without extensive white blood cell infiltration (Fig. 3, Supplementary Fig. S1). This mild phenotype may be due to CFAP221 and CFAP54 both associating with the C1d projection of the CPA (Fig. 1). If disrupting both proteins still largely only perturbs the single complex, the phenotype may not be substantially more severe than single *nm1054* or *Cfap54*^*gt/gt*^ mutants. SPEF2, however, localizes to the C1b projection, so loss of SPEF2 and either CFAP221 or CFAP54 would likely disrupt two different protein complexes, put additional strain on the CPA, and have a more substantial effect on ciliary function. As a result, *nm1054/nm1054;bgh/bgh* and *bgh/bgh;Cfap54*^*gt/gt*^/*Cfap54*^*gt/gt*^ double homozygotes have more severe brain and airway phenotypes, further highlighting genetic interactions between *SPEF2* and *CFAP221* or *CFAP54*. These findings are consistent with previous studies identifying genetic interactions between *RPGR* and *CEP290*^16^ and between SPAG6 and SPAG16L^21^, both due to particularly severe ciliary phenotypes.

Because of the early mortality associated with double homozygosity, combined with the low rate of double homozygote birth (1/16 of offspring from two double heterozygotes), mouse numbers for phenotypic analysis were limited and prevented detailed physiological analysis of ciliary motility. However, histopathological analyses demonstrate severe PCD-associated phenotypes and genetic interactions. Additional cell biological and biochemical studies are required to determine how these protein complexes regulate mammalian ciliary motility, as well as how the mechanisms regulating spermiogenesis differ from those driving motile ciliogenesis. As a result, these studies will enable a better understanding of the genetic and molecular mechanisms underlying PCD inheritance and pathogenesis.

## Methods

### Mice

The *nm1054, bgh,* and *Cfap54*^*gt/gt*^ mouse lines were maintained on the C57BL/6J (B6) and 129S6/SvEvTac (129) backgrounds as previously described^26,27,29^. Each mutation results in loss of transcript or protein expression, and each line was genotyped as previously described^26,27,29^. Since previous studies of the single mutations on B6, 129, and mixed backgrounds indicated that the only phenotype influenced by background strain was hydrocephalus, which was more severe and resulted in early mortality on the B6 background^26,27,29^, all analyses in this study were performed on double heterozygous or double homozygous mice on a mixed background. (B6 × 129)F1 double heterozygotes were generated by crossing a single heterozygote on the B6 background to a different single heterozygote on the 129 background. (B6 × 129)F2 double homozygotes were generated by intercrossing (B6 × 129)F1 double heterozygotes, and occasional (B6 × 129)F3 double homozygotes were generated by intercrossing (B6 × 129)F2 double heterozygotes. Double heterozygous (*nm1054/*+ *;bgh/*+ *, nm1054/*+ *;Cfap54*^*gt/gt*^*/*+ *,* and *bgh/*+ *;Cfap54*^*gt/gt*^*/*+) and double homozygous (*nm1054/nm1054;bgh/bgh, nm1054/ nm1054;Cfap54*^*gt/gt*^*/Cfap54*^*gt/gt*^*,* and *bgh/bgh;Cfap54*^*gt/gt*^*/Cfap54*^*gt/gt*^) animals surviving to 8 weeks of age were used for all analyses at that age or later. Double homozygous animals dying prior to 8 weeks due to phenotype severity were used for histological and immunohistochemical analysis at the age of death when available. Wild type (WT) animals from the same generations were used as controls. Mouse numbers are detailed in Supplementary Table S1. Due to early mortality associated with double homozygotes, it was often difficult to collect or analyze samples, but the pathological phenotype was still assessable in the available samples. Other than male infertility, no sex-specific differences have ever been observed for the phenotypes associated with *nm1054, bgh,* or *Cfap54*^*gt/gt*^ PCD^26–30^. Therefore, male and female mice were pooled for analyses in this study. All experiments involving animals were performed in accordance with the Animal Welfare Act and National Institutes of Health (NIH) policies and were approved by the Sanford Research Institutional Animal Care and Use Committee.

### Histology

Heads were immersion fixed in Bouin’s fixative until the bones were decalcified. Coronal slices were then cut through the maxillary sinuses and the lateral ventricles of the brain. Testes were immersion fixed in 10% buffered formalin. Fixed testes, brain slices, and sinus slices were embedded in paraffin, sectioned, and stained with Hematoxylin and Eosin (H&E) as previously described^28–30^. Stained tissue sections were analyzed by light microscopy using an upright Leica DM6000B microscope and Leica Aperio VERSA slide scanner. Spermatozoa were collected from the epididymis and diluted in phosphate buffered saline (PBS), following which they were spread onto slides, dried, fixed in methanol, and stained with the Camco differential stain kit (Cambridge Diagnostic Products, Inc.) as previously described^29^. Stained spermatozoa were analyzed by light microscopy on an Olympus IX71 inverted microscope.

Because some double homozygous mice died or required euthanasia at earlier ages, thereby resulting in different tissue and organ sizes, severity of brain and sinus phenotypes were quantitatively compared through a scoring system. Severity of the brain phenotype was scored based on the following criteria: 1—no ventricular dilatation; 2—slight dilatation or opening of the ventricle with no visible damage to adjacent tissue; 3—extensive dilatation into the center of the hemisphere with no visible damage to adjacent tissue; 4—extensive dilatation into the center of the hemisphere with damage to adjacent tissue (loss of ependyma, loss of white matter, cortical thinning, and/or hemorrhaging). Severity of the sinus phenotype was scored based on the following criteria: 1—no visible mucus in the maxillary sinus cavity; 2—traces of mucus accumulating in the maxillary sinus cavity; 3—extensive mucus accumulation filling large portions of the sinus cavity; 4—extensive mucus accumulation filling large portions of the sinus cavity with infiltration of neutrophils and/or red blood cells. Statistical significance was determined by one-way ANOVA.

### Immunohistochemistry

Testes, brain slices, and sinus slices were fixed and prepared as described above for histology. The fixed tissues were embedded in paraffin, sectioned, and stained using the BenchMark XT automated slide staining system (Ventana Medical Systems, Inc.) as previously described^28–30^. Primary antibodies included a mouse acetylated tubulin antibody (Sigma Aldrich T6793) at a 1:10,000 dilution, a mouse γ-tubulin antibody (Sigma Aldrich T6557) at a 1:500 dilution, a rabbit SPAG6 antibody (Invitrogen PA5-58389) at a 1:500 dilution, a rabbit RSPH4A antibody (Sigma Aldrich HPA031198) at a 1:100 dilution, and a rabbit DNAI1 antibody (Sigma Aldrich HPA021649) at a 1:300 dilution. Biotin SP-conjugated AffiniPure donkey anti-mouse (Jackson ImmunoResearch 715-065-151) and goat anti-rabbit (Jackson ImmunoResearch 111-065-144) secondary antibodies were both used at a 1:1,000 dilution. The slides were visualized by light microscopy using the upright Leica DM6000B microscope and Leica Aperio VERSA slide scanner, and high magnification images were acquired using the upright Nikon Eclipse Ni-E microscope with a 100 × objective and a Nikon DS-2MV camera.

The Aperio ImageScope software (v12.3.3.7014) was used for quantification of staining intensity on slide-scanned images of sinus sections. Regions of interest were drawn around cilia in fields where the ciliated epithelial cells were clearly in focus. Positivity, which is determined by the number of positive pixels divided by the number of total pixels (positive staining plus negative counter-stain pixels) in a selected region, represents staining intensity and was calculated for each image using the Positive Pixel Count v9 analysis algorithm with default manufacturer input settings. Statistical analysis was performed using GraphPad Prism 6 (v6.02). Outliers were identified using the ROUT analysis (Q = 1%), and any rare data point that was an order of magnitude different from all other values within a given cohort was removed from analysis. Statistical significance was determined by one-way ANOVA.

### Ciliary beat frequency (CBF) analysis

Tracheae were dissected into Medium 199, Hank’s Balanced Salts and equilibrated at room temperature for 1 h, after which they were cut into rings using a Nikon SMZ1000 stereomicroscope. The rings were placed into fresh media and maintained at 28 °C using a thermal plate (Takai Hit TP-110RS05) for consistency throughout the experiment. CBF was analyzed on an Olympus IX71 inverted microscope using the Sisson-Ammons Video Analysis system as previously described^26,27,29,40^. Statistical significance was determined by one-way ANOVA.

## Supplementary information

Supplementary Information

## Data Availability

No large datasets were generated or analyzed during the current study. Representative data are included in this published article (and its Supplementary Information files), and all raw data is available from the corresponding author upon reasonable request.
