# Supplementary material for: Genetic interaction between central pair apparatus genes CFAP221, CFAP54, and SPEF2 in mouse models of primary ciliary dyskinesia
Source: Sci Rep. 2020 Jul 23;10:12337. doi: 10.1038/s41598-020-69359-3 (PMC7378221; doi:10.1038/s41598-020-69359-3)
Supplement: Supplementary file 1 — Supplementary Information [file 41598_2020_69359_MOESM1_ESM.pdf]

TITLE: Genetic Interaction between Central Pair Apparatus Genes *CFAP221, CFAP54,* and *SPEF2* in Mouse Models of Primary Ciliary Dyskinesia

AUTHORS: Casey W. McKenzie[1] and Lance Lee[1,2,*]

AFFILIATIONS:
[1]Pediatrics and Rare Diseases Group, Sanford Research, 2301 E. 60th Street N., Sioux Falls, SD 57104

[4]Department of Pediatrics, Sanford School of Medicine of the University of South Dakota, 1400 W. 22nd Street, Sioux Falls, SD 57105

Supplementary Table 1. Mouse Numbers

Genotype	Total n	Early Mortality[a]	Sinus Histology	Brain Histology	Testis Histology	Sperm Morphology	CBF[b]
WT	16	0	10	7	8	6	11
nm1054/+; bgh/+	14	0	9	5	8	7	12
nm1054/+; Cfap54$^{gt/gt}$/+	15	0	5	5	7	6	10
bgh/+; Cfap54$^{gt/gt}$/+	17	0	10	5	9	5	15
nm1054/nm1054; bgh/bgh	11	11	1	1	0[c]	0[c]	0
nm1054/nm1054; Cfap54$^{gt/gt}$/ Cfap54$^{gt/gt}$	8	1	7	7	4	3	0
bgh/bgh; Cfap54$^{gt/gt}$/ Cfap54$^{gt/gt}$	12	7	8	9	3	2	0

[a]Death prior to 8 weeks, often in the postnatal period preventing collection and analysis of samples
[b]Since so few double homozygous mutants survived to 8 weeks, CBF data is only reported for double heterozygotes and WT controls
[c]No *nm1054/nm1054; bgh/bgh* mice survived to sexual maturity, and the only mouse that survived long enough for tissue collection was female

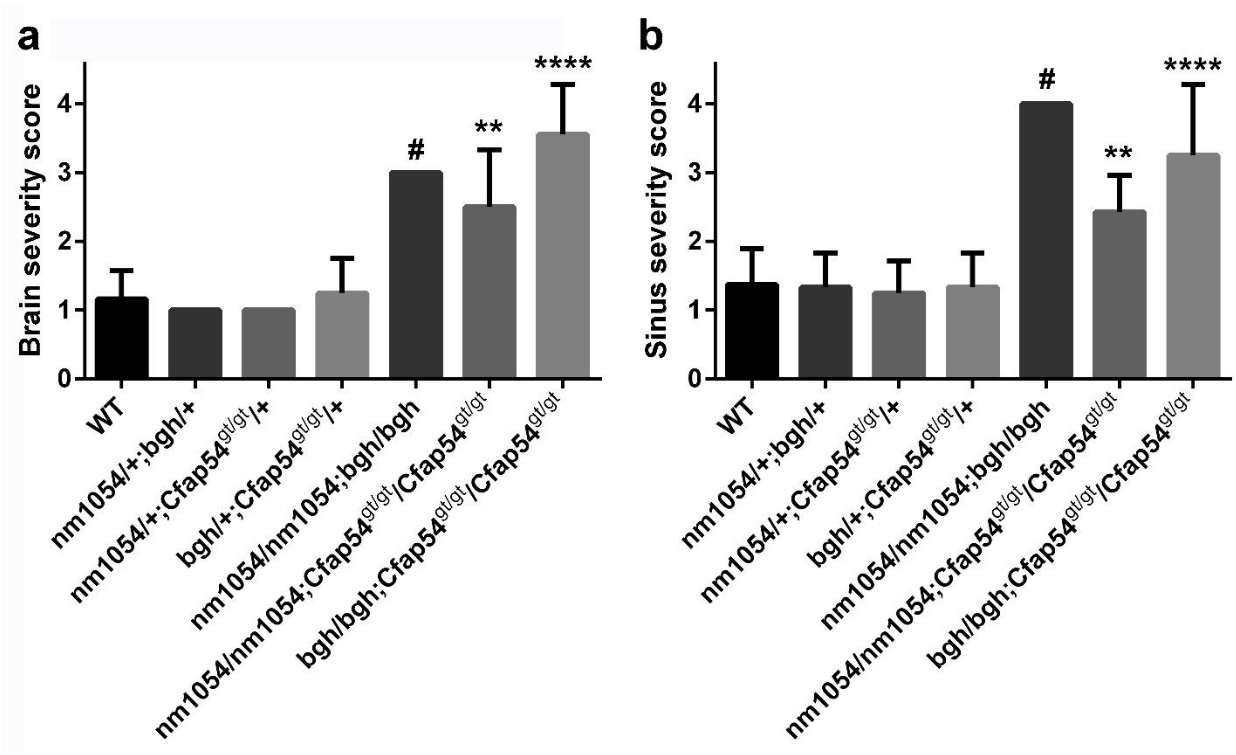

Supplementary Figure 1. Severity scoring for brain and sinus phenotypes. Brain (a) and sinus (b) phenotype severity scores for WT, *nm1054/+;bgh/+*, *nm1054/+;Cfap54*$^{gt/gt}$/+, *bgh/+;Cfap54*$^{gt/gt}$/+, *nm1054/nm1054;bgh/bgh*, *nm1054/nm1054;Cfap54*$^{gt/gt}$/*Cfap54*$^{gt/gt}$, and *bgh/bgh;Cfap54*$^{gt/gt}$/*Cfap54*$^{gt/gt}$ mice. All p values are relative to WT. For brains, p = 0.983 for *nm1054/+;bgh/+*, 0.979 for *nm1054/+;Cfap54*$^{gt/gt}$/+, 0.999 for *bgh/+;Cfap54*$^{gt/gt}$/+, 0.001 for *nm1054/nm1054;Cfap54*$^{gt/gt}$/*Cfap54*$^{gt/gt}$, and < 0.001 for *bgh/bgh;Cfap54*$^{gt/gt}$/*Cfap54*$^{gt/gt}$ mice. For sinuses, p = 0.999 for *nm1054/+;bgh/+*, 0.994 for *nm1054/+;Cfap54*$^{gt/gt}$/+, 0.999 for *bgh/+;Cfap54*$^{gt/gt}$/+, 0.009 for *nm1054/nm1054;Cfap54*$^{gt/gt}$/*Cfap54*$^{gt/gt}$, and < 0.001 for *bgh/bgh;Cfap54*$^{gt/gt}$/*Cfap54*$^{gt/gt}$ mice. Statistical significance was determined by one-way ANOVA. Since only one *nm1054/nm1054;bgh/bgh* mouse survived for tissue collection, no statistical analysis was performed (denoted by #).

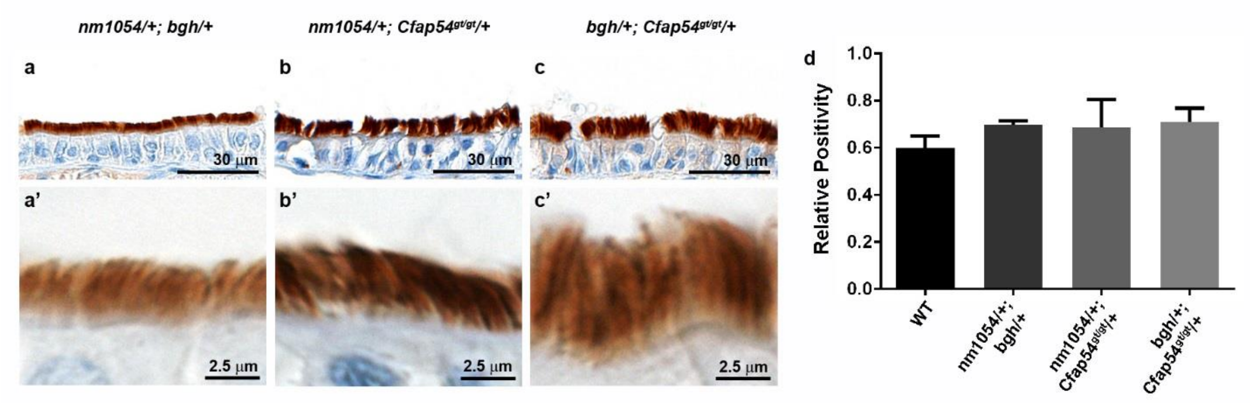

Supplementary Figure 2. Immunohistochemical analysis of double heterozygous sinus epithelial cilia. Sections of *nm1054/+;bgh/+* (a,a'), *nm1054/+;Cfap54*$^{gt/gt}$/+ (b,b'), and *bgh/+;Cfap54*$^{gt/gt}$/+ (c,c') maxillary sinus airway epithelia stained with an antibody to ciliary marker acetylated tubulin. Quantification of acetylated tubulin staining intensity is shown in (d). All p values are relative to WT. $p = 0.166$ for *nm1054/+;bgh/+*, 0.245 for *nm1054/+;Cfap54*$^{gt/gt}$/+, and 0.135 for *bgh/+;Cfap54*$^{gt/gt}$/+ mice. Statistical significance was determined by one-way ANOVA.

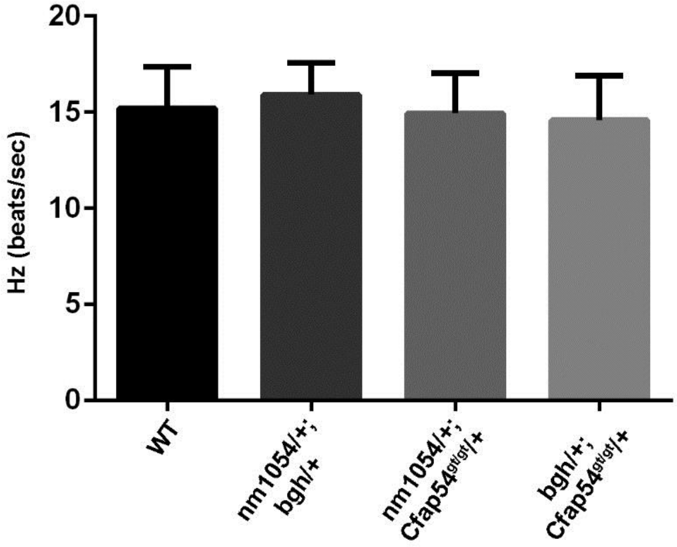

Supplementary Figure 3. Double heterozygous tracheal CBF analysis. Tracheal epithelial CBF from WT, *nm1054/+;bgh/*+, *nm1054/+;Cfap54*$^{gt/gt}$/+, and *bgh/+;Cfap54*$^{gt/gt}$/+ mice in beats per second (Hz) shows no statistically significant difference between WT and double heterozygous levels. p = 0.767 for *nm1054/+;bgh/*+, 0.982 for *nm1054/+;Cfap54*$^{gt/gt}$/+, and 0.799 for *bgh/+;Cfap54*$^{gt/gt}$/+ relative to WT. Statistical significance was determined by one-way ANOVA.

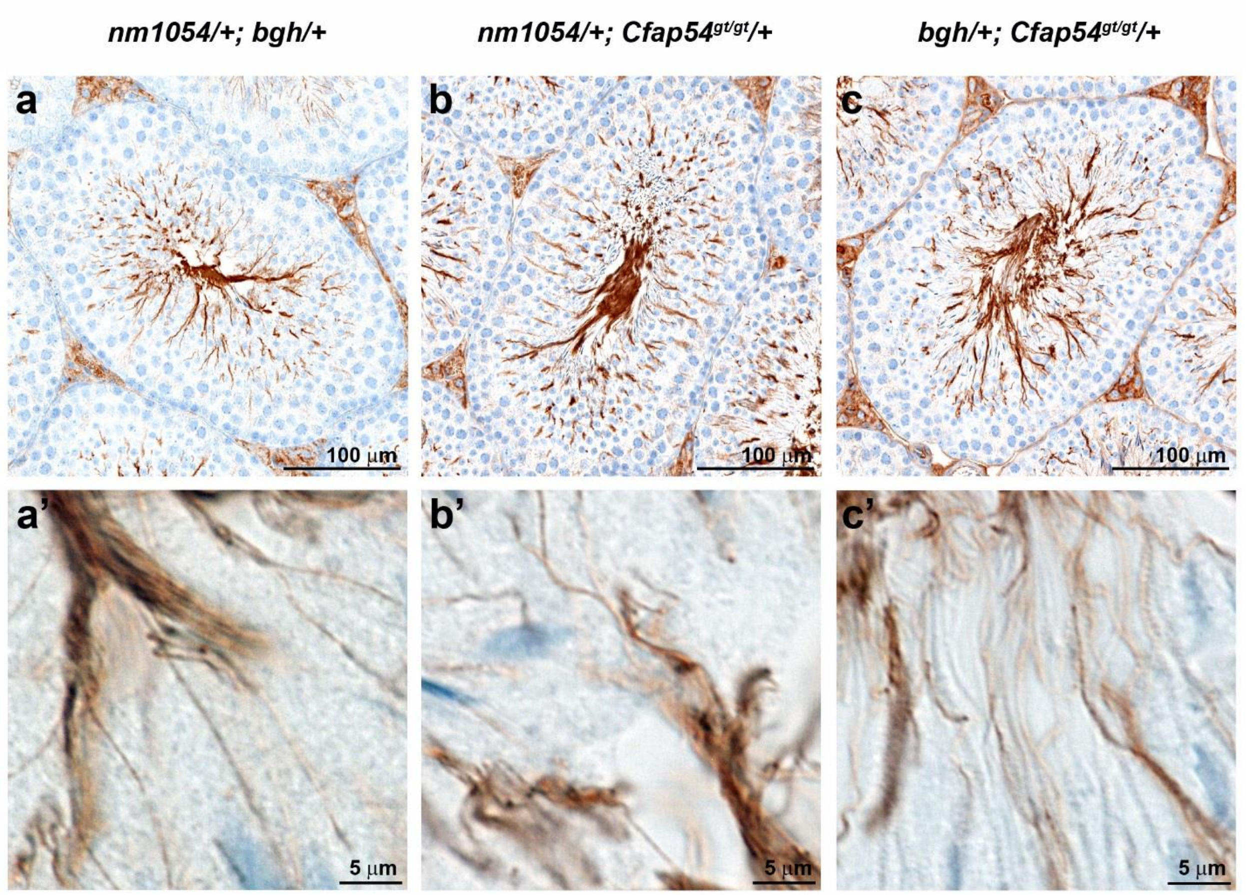

Supplementary Figure 4. Immunohistochemical analysis of double heterozygous testis. Sections of *nm1054/+;bgh/+* (a,a'), *nm1054/+;Cfap54$^{gt/gt}$/+* (b,b'), and *bgh/+;Cfap54$^{gt/gt}$/+* (c,c') testis stained with an antibody to flagellar marker acetylated tubulin, indicating normal flagellar formation and morphology.
